# Nitrogen Dynamics in Soil Fertilized with Slow Release Brown Coal-Urea Fertilizers

**DOI:** 10.1038/s41598-018-32787-3

**Published:** 2018-10-01

**Authors:** Biplob K. Saha, Michael T. Rose, Vanessa N. L. Wong, Timothy R. Cavagnaro, Antonio F. Patti

**Affiliations:** 1 School of Chemistry, Monash University, Clayton, Victoria 3800 Australia; 2 NSW Department of Primary Industries, Wollongbar Primary Industries Institute, Wollongbar, NSW 2477 Australia; 3 School of Earth, Atmosphere & Environment, Monash University, Clayton, Victoria 3800 Australia; 4 The Waite Research Institute and The School of Agriculture, Food and Wine, The University of Adelaide, Waite Campus, PMBI Glen Osmond, South Australia, 5064 Australia

## Abstract

Reducing the release rate of urea can increase its use efficiency and minimize negative effects on the environment. A novel fertilizer material that was formed by blending brown coal (BC) with urea, delayed fertilizer N release in controlled climatic conditions in a glasshouse, through strong retention facilitated by the extensive surface area, porous structure and chemical functional groups in the BC. However, the role of BC as a carrier of synthetic urea and the effect of their interaction with various soil types on the dynamics and mineralization of N remains largely unclear. Therefore, a soil column incubation study was conducted to assess the release, transformation and transportation of N from several different brown coal-urea (BCU) granules, compared to commercial urea. Blending and subsequent granulation of urea with BC substantially increased fertilizer N retention in soil by decreasing gaseous emissions and leaching of N compared to urea alone, irrespective of soil type. The BCU granule containing the highest proportion of BC had lower leaching and gaseous emissions and maintained considerably higher mineral and mineralizable N in topsoil. Possible modes of action of the BCU granules have been proposed, emphasizing the role of BC in enhancing N retention over a longer period of time. The results support the notion that BCU granules can be used as a slow release and enhanced efficiency fertilizer for increasing availability and use efficiency of N by crops.

## Introduction

Nitrogen (N) is one of the most important and limiting nutrients for agricultural crop production systems^1^. As a result, high rates of synthetic N fertilizers are commonly applied to improve agricultural crop production world-wide. Unfortunately, more than 50% of the added N fertilizers are commonly lost through leaching, denitrification and volatilization, resulting in poor fertilizer N use efficiency^2^. The movement and transport of this lost N can cause serious global environmental pollution by contaminating groundwater through nitrate leaching (124–160 kg N ha^−1^ yr^−1^) and by contributing to greenhouse gas emissions (120–143 kg N ha^−1^ yr^−1^) to the atmosphere^3–6^. This lost N is also responsible for economic inefficiency by increasing the cost of agricultural production without contributing to higher yields. From both an economic and an environmental perspective, it is therefore necessary to increase the use efficiency of fertilizer N by reducing its losses via different pathways.

Considerable research efforts have been aimed at developing suitable mitigation strategies to reduce gaseous and leaching losses of N from intensively managed agricultural systems^7–10^. Mitigation strategies, such as using controlled release or enhanced efficiency fertilizers or combined application of organic and inorganic fertilizers to minimize the loss of N for improving its use efficiency by crops have been extensively studied in the last few decades^11,12^. However, the broadacre application of these technologies has not been adopted in intensive farming systems due to the lack of consistency and cost in research findings confirming the efficacy of these strategies^13^.

Granulation of inorganic fertilizers with organic materials is increasingly becoming more popular because of its potential benefits on crop yields and soil health compared to the application of organic material or inorganic fertilizer as a sole nutrient source^14,15^. Application of organo-mineral granules has been shown to reduce nutrient losses from soil and significantly increase yield and N uptake by crops^7,14–16^. Indeed, the depletion of soil organic carbon (SOC) due to intensification of agricultural crop production can be another reason for declining fertilizer N use efficiency^17^. This is because SOC plays an important role in the retention of fertilizer N in soil and limits off-site N losses^18^. Organo-mineral granules supply organic matter simultaneously with added nutrients, which helps to reduce immediate nutrient release due to increased adsorption by organic matter and/or microbial nutrient immobilization^16^. As a result, nutrients are available to plants for longer periods during the plant growing cycle with the implication that less fertilizer needs to be applied to the soil, thereby reducing the potential for environmental pollution and the cost of production^19^.

There is a growing interest in the utilization of brown coal (BC) and BC-derived products in agricultural crop production due to their particular physical and chemical properties^20^. The extensive surface area, porous structure and functional groups of BC^21^ have been demonstrated to increase the prevalence of carboxyl and phenolic functional groups in BC-amended soil^22^, enhanced nutrient retention and uptake^23^, and facilitate cation binding^24^ with minimal effects on microbial activity^25^. It is also evident that the addition of BC alone or in combination with urea can significantly influence the dynamics and mineralization of N in soil^7,8,26,27^. In a recent study it was found that granulation of urea with BC substantially reduced the release rate of fertilizer N compared to urea alone^26^. Multiomics experimental results showed decreased NH_3_ emissions^27,28^, N_2_O emissions and leaching loss of N^7^, whereas increased N_2_O emissions^8,27^ were also reported by the addition of BC alone or in combination with urea. A better understanding of the effect of blending and subsequent granulation of BC with urea on the dynamics and mineralization of N in various soil types is critical to enable the successful use of brown coal-urea (BCU) granules as slow release N fertilizer. In this study, we tested the hypothesis that blending and subsequent granulation of urea with BC reduces N losses by leaching and gaseous emissions, compared with conventional urea fertilizer, in three different soil types.

## Results

### Leaching and Gaseous Loss of N from Soil

**Figure 1:**
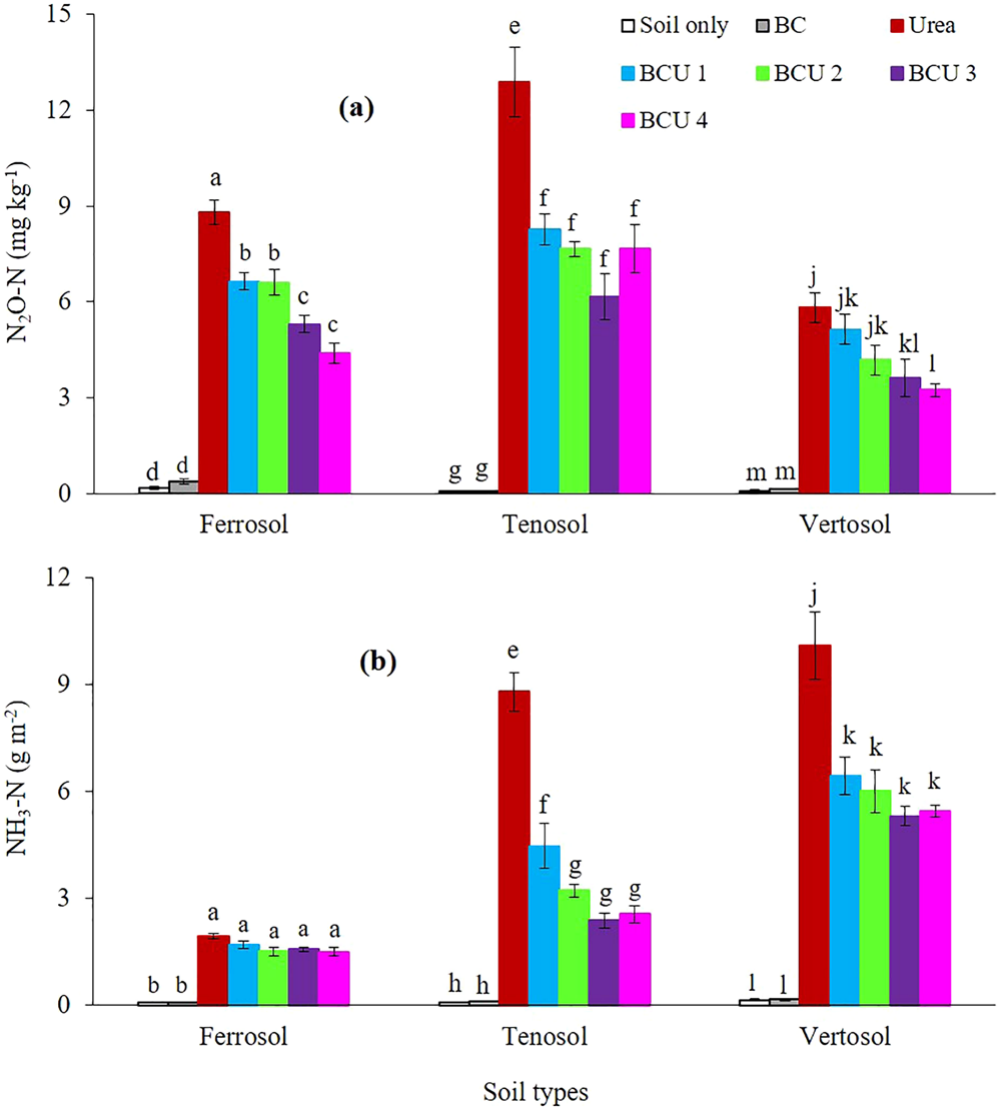
Cumulative N_2_O-N (**a**) and NH_3_-N (**b**) emissions from different soil types (values are mean ± standard error, N = 5). Letters above columns are different if the values are significantly different (*P* < 0.05). Valid statistical comparisons cannot be made between soil types.

**Figure 2:**
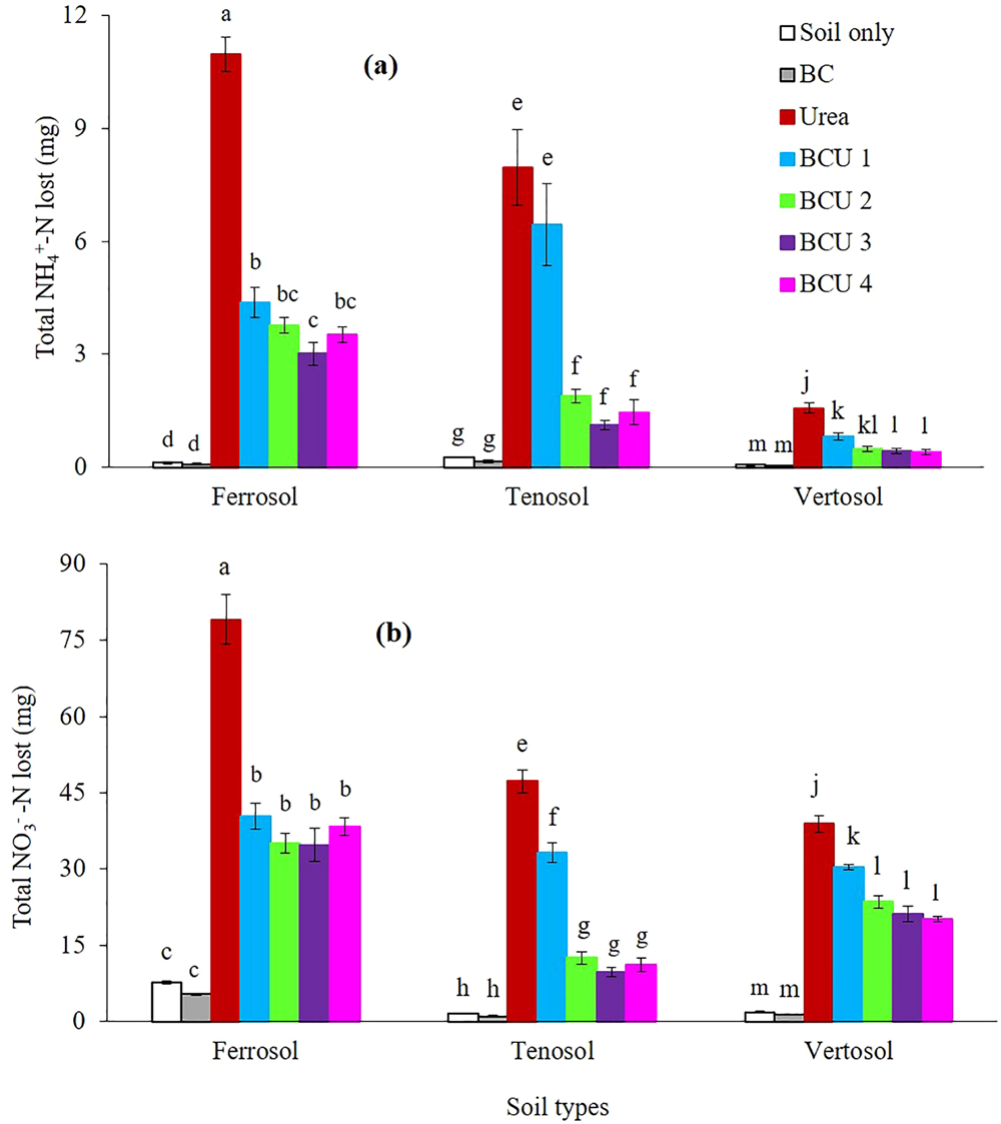
Leaching loss of NH_4_^+^-N (**a**) and NO_3_^−^-N (**b**) from soil (values are mean ± standard error, N = 5). Letters above columns are different if the values are significantly different (*P* < 0.05). Valid statistical comparisons cannot be made between soil types.

Addition of urea and BCU granules significantly increased the gaseous (N_2_O and NH_3_) (Fig. 1) and leaching loss of mineral N (NH_4_^+^-N and NO_3_^−^-N) (Fig. 2) from the columns of all three soil types, compared to columns not receiving any N fertilizer. Compared to urea alone, granulation of urea with BC significantly reduced the amount of mineral N leached out from the different soils in all the leaching events, except the leaching event at 30 days after fertilizer addition (DAF) in the Tenosol, where no significant variations in mineral N concentrations were observed among urea, BCU 1 and BCU 2 (SI Figures S1 and S2). In general, increasing the ratio of BC to N reduced the amount of mineral N lost through leaching. The leaching of mineral N was much higher in the Ferrosol compared to the Tenosol and Vertosol. On average, addition of BCU granules reduced leaching losses of mineral N by 56, 65 and 42% from the Ferrosol, Tenosol and Vertosol, respectively compared to urea fertilized soil.

The daily N_2_O emissions from urea and BCU treated soil reached a peak at 14 days after fertilizer addition (DAF) in the Tenosol and Vertosol whereas in the Ferrosol, N_2_O emissions for the urea treated soil peaked at 3 DAF (SI Figure S3). A dramatic increase in N_2_O emissions was monitored after each leaching event in all soil types. The increased soil moisture and subsequent reduction in air-filled pore space after each leaching event may have triggered the activity of denitrifying microbes and enhanced N_2_O emissions in soil^29,30^. The addition of BCU granules resulted in a significant decrease in daily and cumulative N_2_O emissions compared to straight urea, irrespective of soil type. In general, the granules with higher BC had significantly lower N_2_O emissions from soils compared to the granules with lower BC. Compared to urea, on average, BCU granules suppressed the cumulative N_2_O emissions by 35, 42 and 31% from the Ferrosol, Tenosol and Vertosol, respectively.

A sharp increase in NH_3_ volatilization was measured one day after fertilizer addition and reached its peak rapidly within one week of fertilization in all soils (SI Figure S4). Ammonia volatilization then declined and remained almost constant until the end of the incubation, irrespective of soil type. The addition of BCU granules resulted in a significant decrease in daily and cumulative volatilization loss of NH_3_ from the Tenosol and Vertosol compared to the soil treated with urea only (Fig. 1b). However, no significant variation was found in the NH_3_ volatilization among the urea and BCU treated soils in the Ferrosol. The volatilization loss of NH_3_ was considerably higher in the Vertosol compared to the Tenosol and Ferrosol. The granules with higher BC had significantly lower NH_3_ volatilization from soil compared to the granules with lower BC. Compared to urea, on average, BCU granules suppressed the cumulative NH_3_ emissions by 19, 64 and 42% from the Ferrosol, Tenosol and Vertosol, respectively.

### Mineral N, Mineralizable N, Total N and C Content of the Soil Profile

**Figure 3:**
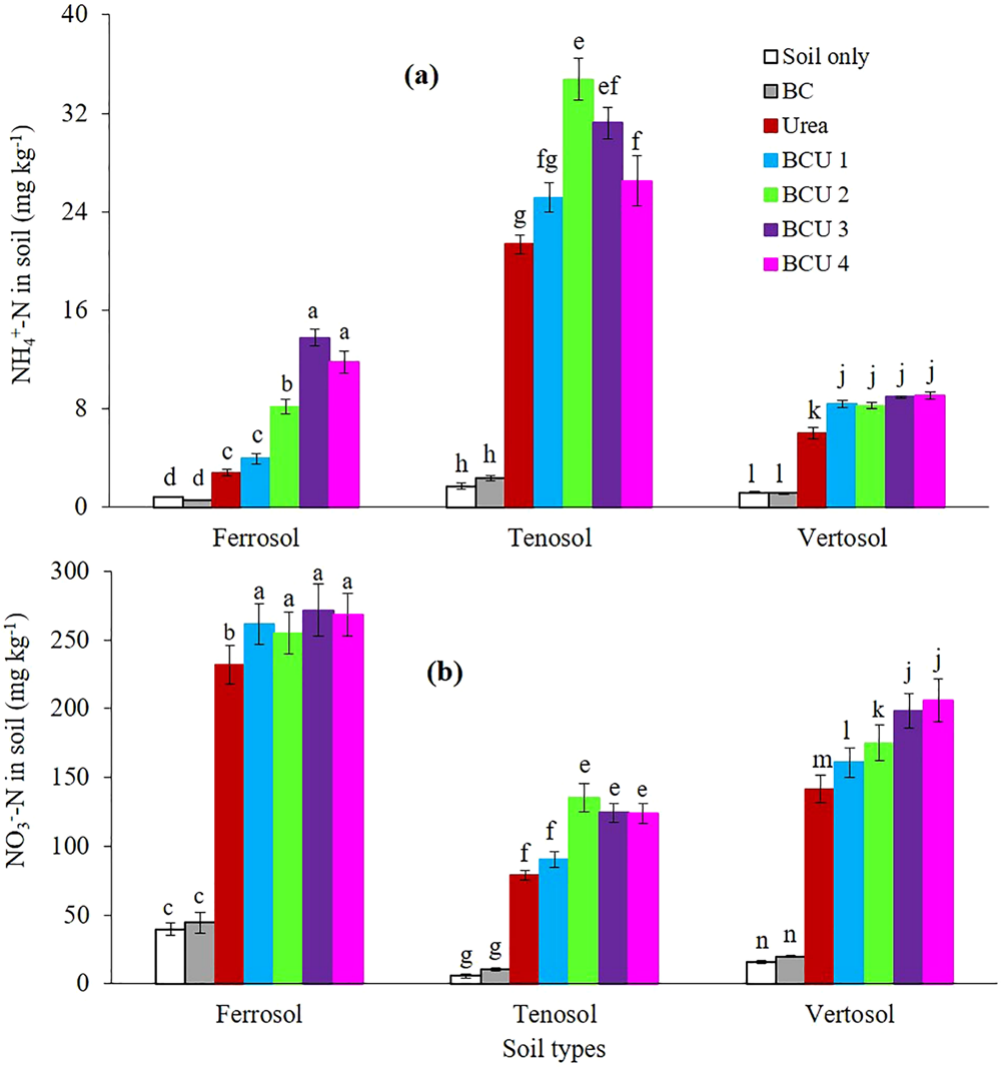
Average NH_4_^+^-N (**a**) and NO_3_^−^-N (**b**) content of soil profile at the end of incubation (values are mean ± standard error, N = 5). Letters above columns are different if the values are significantly different (*P* < 0.05). Valid statistical comparisons cannot be made between soil types.

**Figure 4:**
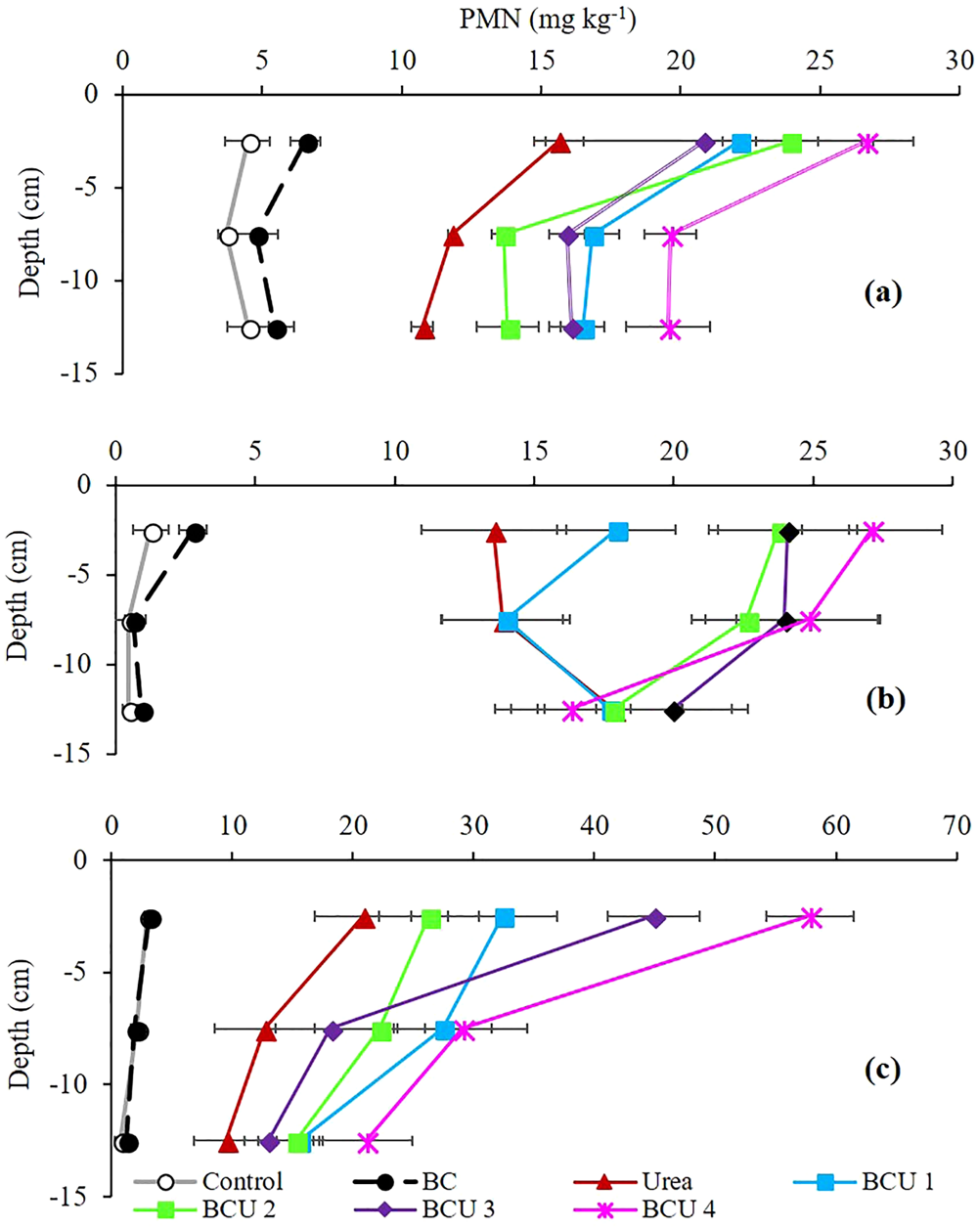
PMN content of the soil profile at the end of incubation in a Ferrosol (**a**), Tenosol (**b**) and Vertosol (**c**) (values are mean ± standard error, N = 5).

Significantly higher amounts of mineral N and potentially mineralizable N (PMN) were measured from the soil fertilized with urea and BCU granules compared to BC and control soil (Figs 3 and 4). Addition of BCU granules maintained significantly higher amounts of mineral and mineralizable N in the top layer (0–5 cm) of the soil compared to the soil treated with urea only (SI Figures S5–7). However, no significant difference in mineral and mineralizable N was found between urea and BCU granule treatments in soil depth from 6 to 15 cm in all the soil types (SI Table S6). In the Ferrosol and Vertosol, the mineral and PMN concentrations decreased with the increase in soil profile depth whereas an opposite trend was noticed in the Tenosol for urea and BCU 1 treatments. Generally, the BCU granules with higher amounts of BC retained higher mineral and PMN in soil than the granules containing lower amounts of BC. Compared to urea, on average, incorporation of BCU granules increased the NH_4_^+^-N content by 54, 37 and 44%, and NO_3_^−^-N content by 14, 49 and 31%, in the Ferrosol, Tenosol and Vertosol, respectively (Fig. 3).

**Figure 5:**
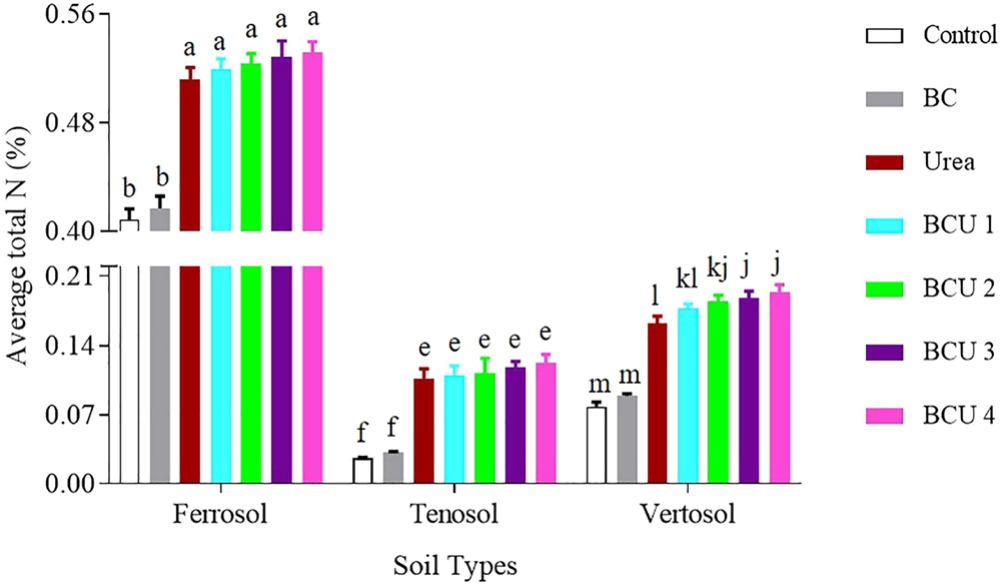
Average total N content of soil profile at the end of incubation (values are mean ± standard error, N = 5). Letters above columns are different if the values are significantly different (*P* < 0.05). Valid statistical comparisons cannot be made between soil types.

Incorporation of urea and BCU granules showed a considerably higher amount of total N in the top (0–5 cm) of the soil column compared to the middle (6–10 cm) and bottom (11–15 cm) layer in all the soil types (SI Figure S8). Significant differences in top soil (0–5 cm) total N were found in the soil fertilized with BCU over urea irrespective of soil type. Application of BCU granules 2, 3 and 4 maintained a significantly higher amount of average total N than the urea treatment only in the Vertosol (Fig. 5). The average total N content was higher in the Ferrosol than the other soils.

**Figure 6:**
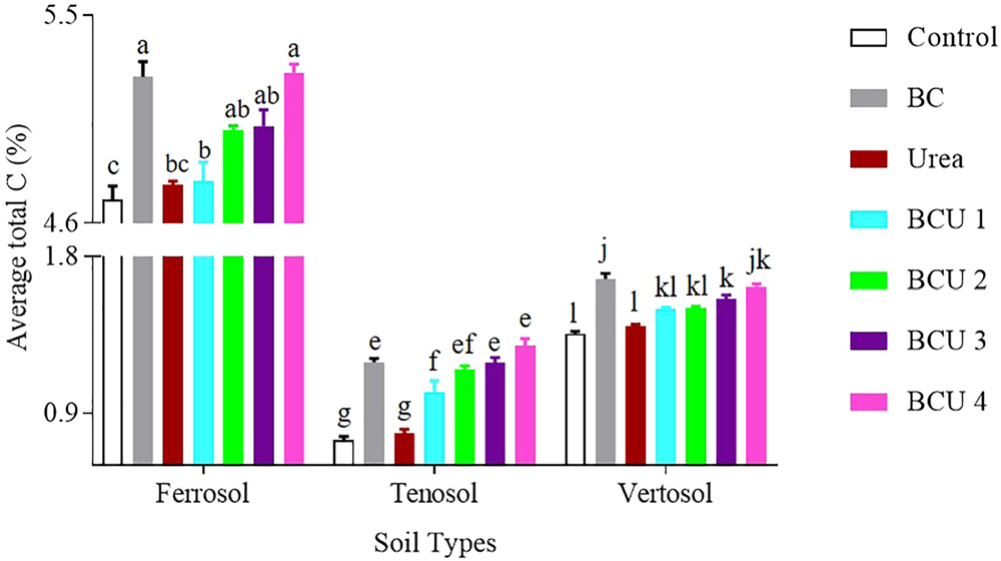
Average total C content of soil profile at the end of incubation (values are mean ± standard error, N = 5). Letters above columns are different if the values are significantly different (*P* < 0.05). Valid statistical comparisons cannot be made between soil types.

Significant changes in the 0–5 cm layer total C content was observed due to the addition of BC and BCU granules in the various soils. A decreasing trend in soil C levels was noticed with the increase in soil depth in all the soil types (SI Tables 2–4). A significantly higher amount of total C was obtained from the BC and BCU granules 3 and 4 treated soils compared to urea and control treatments (Fig. 6). The granules with higher amounts of BC maintained significantly higher amounts of total C in soil compared to the granules with lower amounts of BC. A higher amount of total C was found in the Ferrosol compared to the Tenosol and Vertosol.

### Mass Balance of N

**Figure 7:**
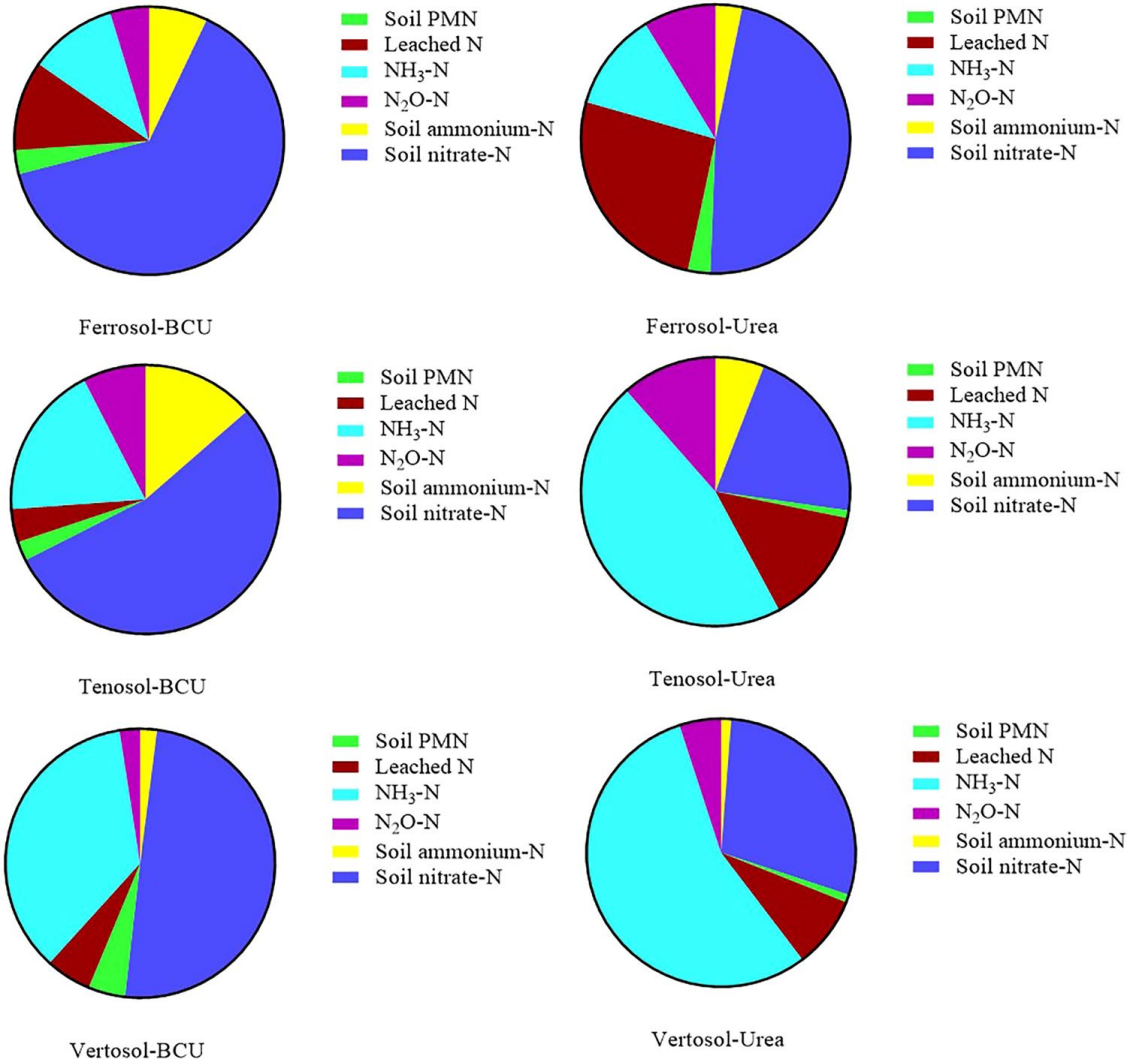
Comparison of measured N balance in various soils treated with urea and BCU (Average of BCU1 to BCU4).

The mass balance of N presented in Fig. 7 provides an overview regarding the effect of BCU granule addition on the dynamics and mineralization of N in various soils. The effect of BCU granules varied substantially depending on soil types. Granulation of BC with urea reduced the leaching loss of mineral N about twice as much as in the Ferrosol and Vertosol. However, in the Tenosol, the reduction in N lost by leaching was around three times compared to urea. The N_2_O emissions were almost double in the urea treated soil compared to BCU granules in all the soil types. Compared to urea, incorporation of BCU granules decreased NH_3_ volatilization loss of N by around 50% in the Tenosol and by about 40% in the Vertosol. In contrast, no significant effect of BCU granules was observed in reducing NH_3_ loss in the Ferrosol. Considerably higher amounts of soil mineral N were determined from the BCU granules amended soil compared to straight urea irrespective of soil types. Addition of BCU granules showed a considerable effect on the mineralization and release of N in soil. Granulation of BC with urea maintained substantially higher ammonium, nitrate and PMN in all the soil types compared to urea alone. The increased PMN is likely to reflect urea that has been retained for longer periods in the soil as a result of being incorporated as BCU material. The ammonium-N was approximately double, and the PMN was around three times higher, in BCU amended soil *cf*. to the urea treated soil. The higher mineral-N in the BCU treated soil indicated slow and steady release of urea-N. Moreover, the substantially higher PMN in the BCU amended soil indicated that still more mineral-N was available for mineralization which could be available to plants over a longer period. A ^15^N tracer experiment will be performed to complete the N mass balance and to calculate the total recovery of fertilizer-N from soil.

## Discussion

Blending of BC with urea can potentially decrease losses of N while simultaneously adding N to the soil profile. The lower NH_4_^+^-N concentration in leachates obtained from the BCU granules treated soil might be due to its adsorption on the BC, which has an extensive porous surface area and high cation exchange capacity (76.3 cmol_c_ kg^−1^) due to the presence of a large number of acidic functional groups^26^. In addition, soil incorporation of BC has been shown to decrease the release rate of urea-N by reducing urea hydrolysis through the inhibition of urease enzyme activity in soil^26,31^. The lower availability of NH_4_^+^ may also decrease the nitrification of NH_4_^+^ to NO_3_^−^ resulting in lower NO_3_^−^-N concentration in leachates obtained from BCU treated soil compared to urea alone, as has been previously observed^7,8^. The lower leaching loss of mineral N from BCU granules containing the higher amount of BC emphasizes the role of BC in N retention. The leaching loss of mineral N was considerably higher in the Ferrosol compared to the Tenosol and Vertosol (Fig. 2). The Ferrosol is dominated by iron and aluminium (oxy)hydroxides which are variable charge minerals (magnetite, goethite, gibbsite etc.), unlike the 2:1 aluminosilicates which are permanent charge minerals (smectite, vermiculite etc.) likely to be present in the Vertosol. At low pH, the variable charge minerals are predominantly positive which may reduce the potential for NH_4_^+^ adsorption. Ammonium ions usually compete with other cations (Fe^2+^, Fe^3+^, Al^3+^, Ca^2+^, K^+^ and Na^+^) for exchange sites in soil. Therefore, an increase in Fe^2+^, Fe^3+^ and Al^3+^ concentration may displace the colloidal-bound NH_4_^+^ from the soil matrix resulting an increased leaching in the Ferrosol compared to other soils. A similar finding was reported by Kithome, Paul^32^ who found a very low NH_4_^+^ adsorption by zeolite, silicate clay with similar properties to common soil silicate clay minerals, in an acidic solution (pH 4.0).

The soil N_2_O emissions are largely influenced by both the properties of BC and soil. However, the mechanisms by which BC decreased N_2_O emissions are still not clear. The reduction in N_2_O emissions from the BCU amended soil could be the result of slower release of urea-N and reduced availability of NO_3_^−^ during the incubation period. This is in good agreement with previous work in which it was found that granulation of BC with urea significantly decreased N_2_O emissions from soil compared to urea^7^. Incorporation of BCU granules to soil can potentially alter the dynamics and mineralization of N by changing soil moisture, pH and C/N ratio of the soil^26^ (SI Table S4). The acidic pH (4.5) of BC may be responsible for the decreased pH in soil fertilized with BCU granules (SI Table S5), which might have constrained the activity of denitrifiers and thereby decreased the N_2_O emissions^33^. Soil incorporation of lignite may also enhance microbial activity by increasing soil moisture content and providing additional organic C as substrate for microbial colonization and food. The increased microbial population and enhanced microbial immobilization of the available urea-N could be an alternative mechanism of less N_2_O production in BCU amended soil.

The results of our study support the findings of Sun, Bai^27^ and Chen, Sun^28^ who also reported that soil incorporation of lignite decreased NH_3_ emissions by 60–68% from cattle manure amended pens compared to control pens where no lignite was added. The inhibition of urease enzyme activity in BCU amended soil may be the main mechanism of decreasing volatilization loss of NH_3_^26^. Alternatively, the decline in pH due to the addition of extra BC may be the cause of lower volatilization in BCU amended soil. In addition, adsorption of NH_3_-N on the porous structure of BC can be an alternate mechanism of reducing volatilization loss of NH_3_ in soil^34,35^.

Among the various soil types, the volatilization loss of N was substantially lower in the Ferrosol than the Tenosol and Vertosol (Fig. 1b). The lower NH_3_ volatilization in the Ferrosol is likely to be the result of the low soil pH (pH = 4.69) because soil pH is one of the most important factors controlling volatilization loss of N in soil (SI Table S5). Cevallos, Correa^36^ observed that most NH_3_ volatilization occurs at soil pH > 7.0, supporting the results of our study where NH_3_ volatilization was substantially higher in the Tenosol (pH = 7.24) and Vertosol (pH = 8.32). The higher calcium carbonate (CaCO_3_) content could be another reason for increased NH_3_ volatilization in the Vertosol. Ammonium reacts directly with CaCO_3_ to produce ammonium bicarbonate or ammonium carbonate, both of which are unstable and rapidly break down to produce NH_3_ gas, CO_2_ gas, water and Ca^2+^. Both the alkaline soil pH and high CaCO_3_ content induced the conversion of NH_4_^+^ to NH_3_ gas as well as increased volatilization loss of NH_3_ in the Vertosol compared to other soils. Pesek, Stanford^37^ also reported that the volatilization loss of N is positively correlated with CaCO_3_ content and pH of soil.

Substantially higher amounts of mineral and PMN in BCU amended soil might be the result of strong and prolonged retention of urea-N by BC and reduced N losses through leaching and gas emissions. Similarly, the role of increased numbers of cation exchange sites and surface area of BC may result in improved N retention, explaining the higher mineral-N content in soil fertilized with BCU granules containing higher amounts of BC (SI Figure S9). Moreover, the higher PMN content in BCU amended soil indicated that still there was some N retained by BC which may become available for mineralization at a later stage. A similar mechanism was also proposed by Christl, Knicker^38^ who reported that humic acids can incorporate N into their structures either directly through chemical reactions or indirectly through microbial activities and subsequent decomposition of the microbial biomass.

The effect of BCU granules on the dynamics and mineralization of N in soil was also largely influenced by the properties of the different soils used in this study. The soil properties, especially organic matter content, clay content, pH and texture, play an important role in the mineralization, transport and movement of N in soil. The organic matter and clay content of the Ferrosol and Vertosol was much higher than the Tenosol. The higher amount of mineral and mineralizable N in the Ferrosol and Vertosol may be due to higher organic matter and clay content, which favored higher and prolonged N retention via increased cation exchange sites compared to the Tenosol. Furthermore, the Ferrosol had the highest concentration of organic C suggesting high soil organic matter content, and therefore, a greater retention of soil nutrients (Table 1), including N. Increased retention of nutrients can occur through the formation of organo-mineral complexes which are more stable and less likely to be lost via leaching or volatilization. Both NH_4_^+^ and NO_3_^−^ can be incorporated into these organo-mineral complexes.

**Table 1:** Physical and chemical properties of soils.

Property	Ferrosol	Tenosol	Vertosol
USDA soil order	Oxisol	Entisol	Vertisol
Texture	Clay loam	Sandy loam	Clay loam
Bulk density (g cm^−3^)	1.31	1.41	1.34
pH (Water)	4.69	7.23	8.32
Total carbon (% w/w)	4.6	0.77	1.31
Total nitrogen (% w/w)	0.54	0.13	0.24
Ammonium nitrogen (mg kg^−1^)	6.1	6.1	20.6
Nitrate nitrogen (mg kg^−1^)	140	2.2	7.7
Phosphorus (Colwell) (mg kg^−1^)	39	10	39
Exchangeable calcium (cmol_c_ kg^−1^)	7.5	4.57	30.8
Exchangeable magnesium (cmol_c_ kg^−1^)	1.3	0.73	6.4
Exchangeable potassium (cmol_c_ kg^−1^)	0.30	0.24	2.18
Extractable sulfur (mg kg^−1^)	79.5	2.7	1.8
DTPA-iron (mg kg^−1^)	394	44	24
Extractable aluminium (mg kg^−1^)	61	2	2

In conclusion, granulation of BC with urea influenced the distribution, bioavailability and ultimate fate of N in soil when compared to urea alone. Granulation of urea with BC decreased the release of fertilizer-N and substantially increased mineral and PMN in soil by decreasing its gaseous and leaching losses. As a result, fertilizer N will theoretically be available to crop plants over a longer time period. We hypothesize that this will lead to increased fertilizer N use efficiency as well as potentially reducing the required application rates of N fertilizers. The soils responded differently to the addition of different BCU granules. The BCU granules were more efficient in Ferrosol and Tenosol for maintain better N by reducing its gaseous losses. The granules containing higher proportions of BC had lower leaching and gaseous emissions and maintained higher mineral N in soil, emphasizing the role of BC in better N retention. Based on the overall results, BCU granules 2 and 3 could be considered as most suitable for slow release and enhanced efficiency N fertilizer for future work. Overall, these findings provide mechanistic support for the development of BCU granules as an enhanced efficiency and slow release N fertilizer for better N retention in the plant-soil system over a longer period of time.

## Materials and Methods

### Site and Soil Description

Three sequential soil column incubation experiments were carried out in the glasshouse of the Plant Science Complex, Monash University, Clayton, Victoria, 3800, Australia. Three contrasting soils were evaluated in this study. The soils were selected based on clear differences in soil properties, including pH, clay content and iron oxide content. The first, a Ferrosol, was collected from an experimental field of Department of Primary Industries (DPI), Wollongbar Research Institute, New South Wales (28°48′50″S and 153°23′51″E) (Humid subtropical climate), the second, a Tenosol, from a farmer’s field in the Wimmera (37°16′37″S and 143°16′49″E) (Semi-arid to sub-humid climate), Victoria and the third, a Vertosol, from an experimental field of Department of Primary Industries (DPI), Horsham Research Institute, Victoria (36°43′8″S and 142°11′46″E) (Semi-arid climate), Australia^39^. Ten soil samples were collected for each soil type from a depth of 0–15 cm in the same cropping season. The collected soil samples were mixed, air dried, homogenised by sieving to less than 0.2 cm, and stored for the incubation study. The physical and chemical properties of soils are presented in Table 1.

### Incubation Experiment

Four brown coal-urea (BCU) granules previously described and characterized^26^, were tested in the soil column incubations. A brief description of different BCU granules with their total C and N contents are listed in Table 2. BCU 1 granule contained the highest %N (21.45%) and lowest %C (39.81%) and BCU 4 granule containing the lowest %N (5.74%) and highest %C (53.83%). A two-month incubation of soil samples was undertaken in polyvinyl chloride (PVC) tubes of 15 cm height and 4.25 cm radius with a volume of 851 cm^3^. The equivalent of 1,115; 1,200 and 1,141 g dry soil was added to each PVC tube to match with the field soil bulk density of 1.31, 1.41 and 1.34 g cm^−^³ for the Ferrosol, Tenosol and Vertosol, respectively. Thirty five PVC columns for each soil type were covered at one end with nylon mesh and then packed with acid washed sand (1 cm depth) and overlying soil. Deionized water was added to maintain the soil moisture content at 60% water-filled pore space (WFPS) throughout the incubation period. The soil was pre-incubated at 22 ± 1 °C for one week to restore microbial activity. After this, the top (0–5 cm) of each soil column was uniformly amended with urea, BCU 1, BCU 2, BCU 3 and BCU 4. The amount of N applied was the same for all treatments, at the rate of 250 mg N kg^−1^ soil, either from granulated urea alone or one of the BCU granules. Two additional treatments were also included: one contained granulated raw BC at a rate equivalent to the BCU 4 and an un-amended control. Each experiment was laid out following a completely randomized design with five replicates. During the incubation period, three leaching events occurred: at days 15, 30, and 45 after treatment application. At each leaching event, soils were adjusted to 60% WFPS and then leached out with approximately 150 mL of deionized water. Leachate was collected in 50 mL plastic containers and then frozen for future analysis of mineral N (SI Table S1). Emissions of NH_3_ and greenhouse gas (N_2_O) were sampled separately at days 1, 3, 6, 10, 14, 16, 29, 31, 44 and 46 days after fertilizer addition (DAF) using a static closed chamber^29^. The NH_3_ volatilization was measured using polyurethane foam absorbers^40^. The headspace concentration of N_2_O was measured at three time events (0, 30, and 60 min after closing the PVC tubes) during each measuring day and N_2_O concentrations were analyzed using an Agilent 7890 A gas chromatograph (GC). At the end of the incubation, the soil columns were dissected into three 5 cm sections. The depth of the different soil profile layers were: top (0–5 cm), middle (6–10 cm) and bottom (11–15 cm). Soil was removed from each section and taken to the laboratory for chemical analysis. The details of ammonia volatilization and greenhouse gas determination, and chemical analysis of soil and leachate can be found in the Supporting Information (SI).

**Table 2:** Crush strength, C and N content of BCU granules.

Granules	C:N	Crush strength (kg)	C content (%)	N content (%)
Brown coal-urea 1 (BCU 1)	1.8	4.75	40	22
Brown coal-urea 2 (BCU 2)	2.7	6.69	46	17
Brown coal-urea 3 (BCU 3)	5.4	2.38	49	9
Brown coal-urea 4 (BCU 4)	10.8	0.42	54	5

### Statistical Analysis

The statistical analyses were performed using statistical software package IBM SPSS, version 20 (SPSS IBM, 2010). All tests of significance were carried out at *P* < 0.05. The normality testing of the data was conducted using the Kolmogorov-Smirnov goodness of fit test and equality of variances was tested using the modified Levene’s test. Some data were not normally distributed and showed unequal variance. Those data were log or Ln transformed before performing analysis of variance (ANOVA). One way ANOVA was performed and the multiple comparisons among the different treatments were done using a Tukey test (SI Table S6). In all the analysis, soil was not considered as factor because the experiments were conducted sequentially.

## Electronic supplementary material

Supporting Information
