# Supplementary material for: Nitrogen Dynamics in Soil Fertilized with Slow Release Brown Coal-Urea Fertilizers
Source: Sci Rep. 2018 Oct 1;8:14577. doi: 10.1038/s41598-018-32787-3 (PMC6167360; doi:10.1038/s41598-018-32787-3)
Supplement: Supplementary file 1 — Supporting Information [file 41598_2018_32787_MOESM1_ESM.docx]

# Nitrogen dynamics in soil fertilized with slow release brown coal-urea fertilizers

**Biplob K. Saha^1^, Michael T. Rose^2^, Vanessa Wong^3^, Timothy R. Cavagnaro^4^, Antonio F. Patti^1,*^**

^1^School of Chemistry, Monash University, Clayton, Victoria, 3800, Australia

^2^NSW Department of Primary Industries, Wollongbar Primary Industries Institute, Wollongbar, NSW 2477, Australia

^3^School of Earth, Atmosphere & Environment, Monash University, Clayton, Victoria, 3800, Australia

^4^The Waite Research Institute and The School of Agriculture, Food and Wine, The University of Adelaide, Waite Campus, PMBI Glen Osmond, South Australia, 5064, Australia

*Corresponding author. Email: tony.patti@monash.edu

**Supporting Information**

Total pages: 22

Number of Figures: 9

Number of Tables: 6

| **SI. No.** | **Title** | **Page** |
| --- | --- | --- |
| **1.** | Leachate analysis | 3 |
|  | Table S1. Volume of lechate collected from soil column | 4 |
|  | Figure S1. Ammonium concentration in leachates | 5 |
|  | Figure S2. Nitrate concentration in leachates | 6 |
| **2.** | **Measurement of N_2_O emissions** | 7 |
|  | Figure S3. Daily N_2_O-N emissions from Ferrosol (A), Tenosol (B) and Vertosol (C) | 8 |
| **3.** | **Measurement of NH_3_ emissions** | 9 |
|  | Figure S4. Daily NH_3_-N emissions from Ferrosol (A), Tenosol (B) and Vertosol (C) | 10 |
| **4.** | **Soil analysis** | 11 |
|  | Figure S5. Ammonium-N content of soil profile | 12 |
|  | Figure S6. Nitrate-N content of soil profile | 13 |
|  | Figure S7. Average PMN content of soil profile | 14 |
|  | Figure S8. Total N concentration of soil | 15 |
|  | Table S2. Total C concentration of Ferrosol at various depth | 16 |
|  | Table S3. Total C concentration of Tenosol at various depth | 17 |
|  | Table S4. Total C concentration of Vertosol at various depth | 18 |
| **5.** | Post-harvest soil pH | 19 |
|  | Table S5. Effect of BCU blends addition on the soil pH | 20 |
|  | Table S6. *P*-value results from one-way ANOVAs for response variables | 21 |
| **6.** | Figure S9. Mechanistic understanding of the effect of BCU and urea on the N dynamics | 22 |

# Leachate Analysis

Leachates were filtered through Advantech filter paper 42 prior to analysis for mineral N species by spectrophotometry in microplate formats using plate reader. Ammonium ion concentrations were quantified by reacting with salicylate and hyphochlorite in a buffered alkaline solution containing sodium nitroprusside as a reductant ^1^. Nitrate was determined by reduction using vanadium (III) combined with detection by acidic Griess reaction ^2^.

Table S1. Volume of leachate collected during different leaching events from soil columns (values are mean ± standard error, N = 5).

| Treatments | Ferrosol | | | Tenosol | | | Vertosol | | |
| --- | --- | --- | --- | --- | --- | --- | --- | --- | --- |
|  | L1 | L2 | L3 | L1 | L2 | L3 | L1 | L2 | L3 |
| Control | 35.0±2 | 33.0±3 | 25.8±2 | 34.8±2 | 36.4±1 | 36.2±2 | 27.4±2 | 27.8±2 | 36.4±1 |
| BC | 30.2±3 | 29.2±2 | 21.6±2 | 30.1±3 | 34.2±2 | 33.6±3 | 23.8±2 | 20.2±1 | 32.4±1 |
| Urea | 41.0±1 | 31.0±2 | 30.8±2 | 33.2±2 | 37.4±2 | 35.8±3 | 24.4±1 | 24.0±3 | 37.0±3 |
| BCU 1 | 37.4±1 | 31.0±3 | 28.0±1 | 29.2±3 | 34.1±2 | 33.6±2 | 22.2±2 | 23.2±2 | 32.4±2 |
| BCU 2 | 29.0±3 | 30.4±2 | 27.2±2 | 25.2±3 | 30.2±4 | 30.4±2 | 20.8±1 | 20.2±2 | 28.2±2 |
| BCU 3 | 29.4±1 | 26.2±1 | 29.4±4 | 23.6±1 | 31.5±3 | 32.2±3 | 16.8±2 | 22.2±2 | 36.4±2 |
| BCU 4 | 31.8±4 | 29.0±2 | 29.4±2 | 24.2±3 | 29.2±3 | 30.5±3 | 18.8±3 | 22.0±2 | 31.4±2 |

DAF= Days after fertilizer addition, L1= Leaching at 15 DAF, L2= Leaching at 30 DAF and L3= Leaching at 45 DAF


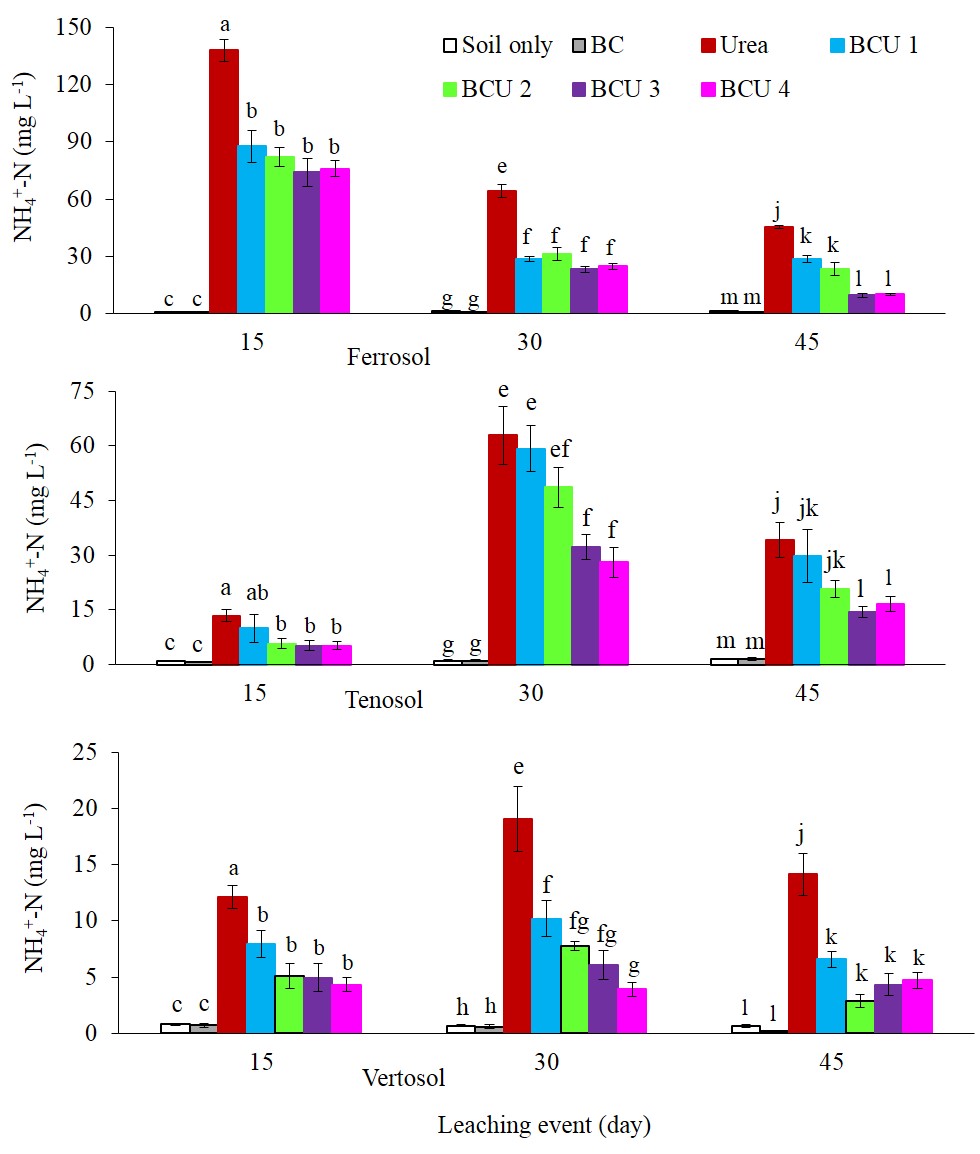
Figure S1. Ammonium concentration in leachates at different leaching events in different soils (values are mean ± standard error, N = 5). Letters above columns are different if the values are significantly different (*P*<0.05). Valid statistical comparisons cannot be made between soil types.


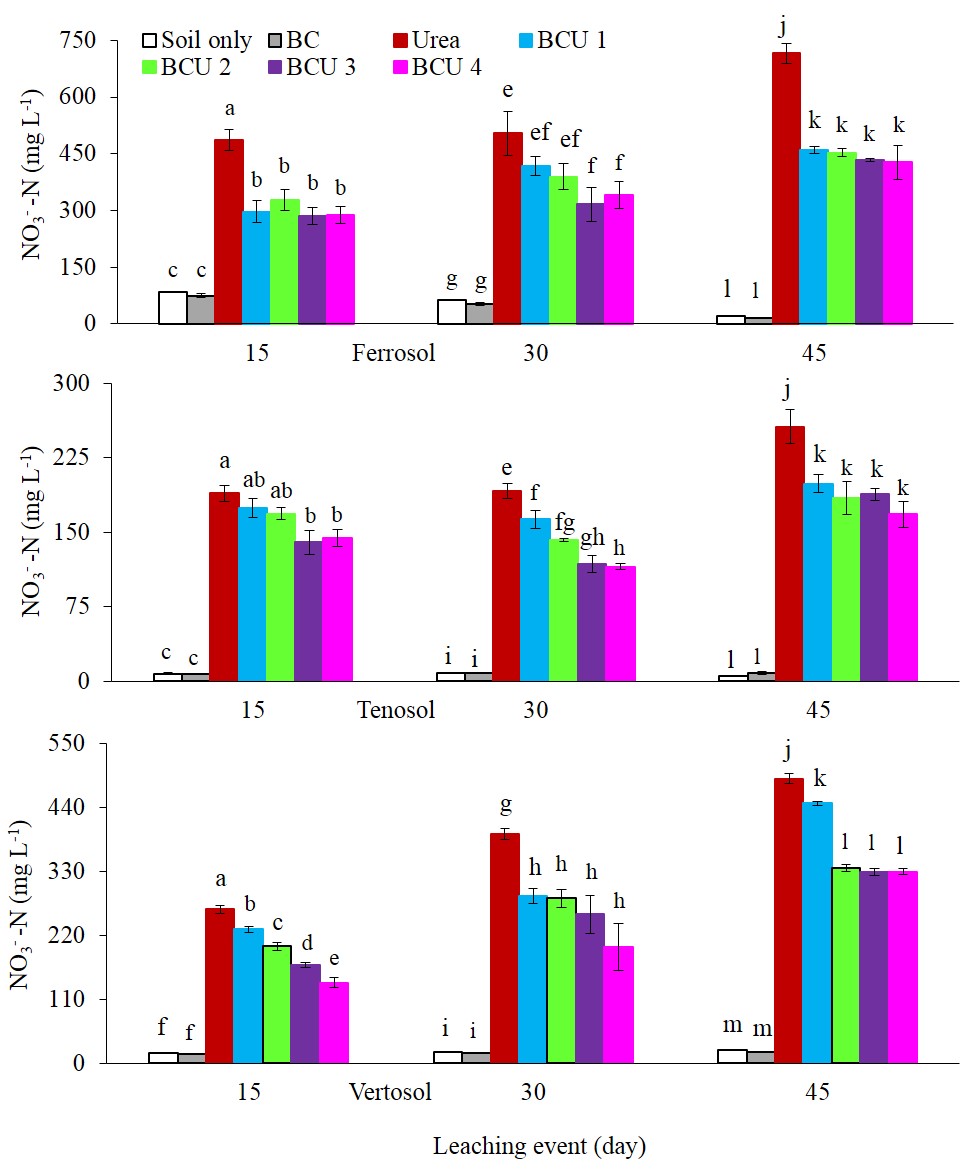


Figure S2. Nitrate concentration in leachates at different leaching events in different soils (values are mean ± standard error, N = 5). Letters above columns are different if the values are significantly different (*P*<0.05). Valid statistical comparisons cannot be made between soil types.

# Measurement of N_2_O Emissions

The PVC tubes were closed with an air tight lid having a rubber septum with a headspace volume of 637 mL for the measurements of N_2_O flux. The headspace concentration of N_2_O was measured at three time events (0, 30, and 60 min after closing the PVC tubes) during each measuring day. At each measurement, a 12 mL gas sample was withdrawn from the headspace of the PVC tubes by an air tight syringe (SGE, 25MDR-LL-GT). The gas sample was then transferred into a 12 mL air tight glass vial which was pre-evacuated and flushed with argon and then re-evacuated. The gas sample was collected in the glass vial and the N_2_O concentration was measured within one week. Gas samples were analyzed for N_2_O using an Agilent 7890A gas chromatograph (GC) fitted with a Gerstel MPS autosampler. The N_2_O concentration was determined with the help of a calibration curve prepared from the reference gas with a known concentration of 1 ppm. Linear interpolation of the gas concentrations were used to calculate the flux and cumulative N_2_O according to the method and equations detailed by Van Zwieten, Kimber ^3^.


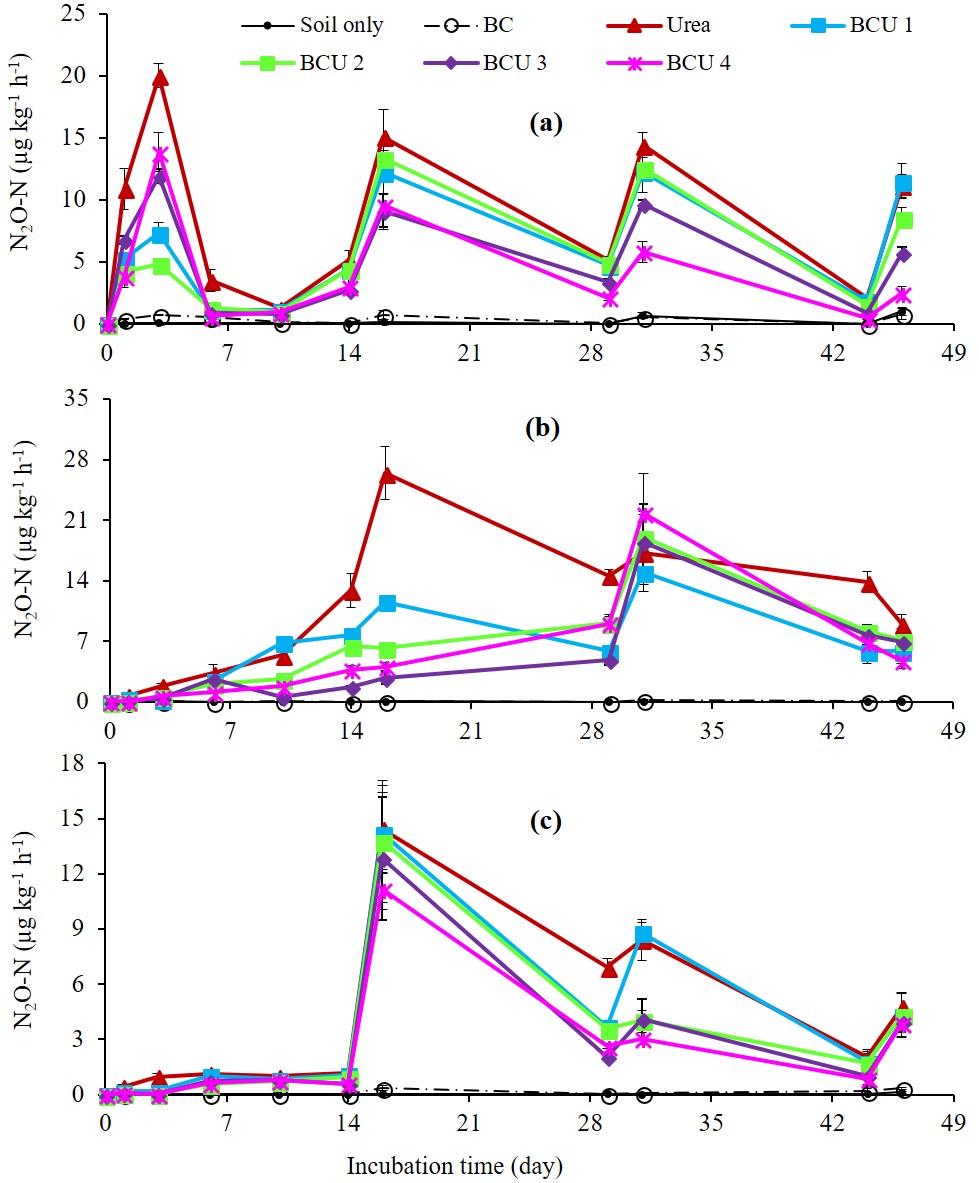


Figure S3. Daily N_2_O-N emissions from Ferrosol (a), Tenosol (b) and Vertosol (c) (values are mean ± standard error, N = 5).

# Measurement of NH_3_ Emissions

The NH_3_ volatilization was measured using polyurethane foam absorbers. The absorbers were placed into plastic petri dish and soaked with 20 mL of 0.05 M sulphuric acid solution. The NH_3_ emissions were measured for 12 h during each sampling event. To prevent contamination with environmental NH_3_, the absorbers were maintained in plastic bags until installation on the PVC tubes. Above each PVC tube one absorber was installed at the top. The petri dishes on the upper side of the absorbers prevented capture of NH_3_ from atmosphere. After this, the absorbers were removed and deep-frozen in plastic bags immediately. At the end of the experimental period, the absorbers were disassembled and components were washed with deionized water. For each absorber, the petri dish was washed with approximately 30 mL of water, using a wash bottle, above the foam placed in a Buckner funnel attached to a Kitassato and a vacuum pump. For a complete removal of the acidic solution, the absorber was washed a second time with deionized water. The solution collected in the Kitassato was then transferred to a volumetric flask and the final volume was made up to 100 mL by adding deionized water. The NH_4_^+^-N concentration was determined colorimetrically by reacting with salicylate and hyphochlorite in a buffered alkaline solution contain sodium nitroprusside as a reductant ^1^. The NH_3_ flux (mg N m^-2^ h^-1^) was then calculated using the equation of Singh, Saggar ^4^.

$$Ammonia flux=\frac{C\times V}{a\times D} (1)$$

Where, C is NH_3_ concentration in the acid trap (mg dm^-3^); V is the volume of acid (dm^3^); *a* is total cross-section area (m^2^) of soil column and D is duration (h) of each sampling.


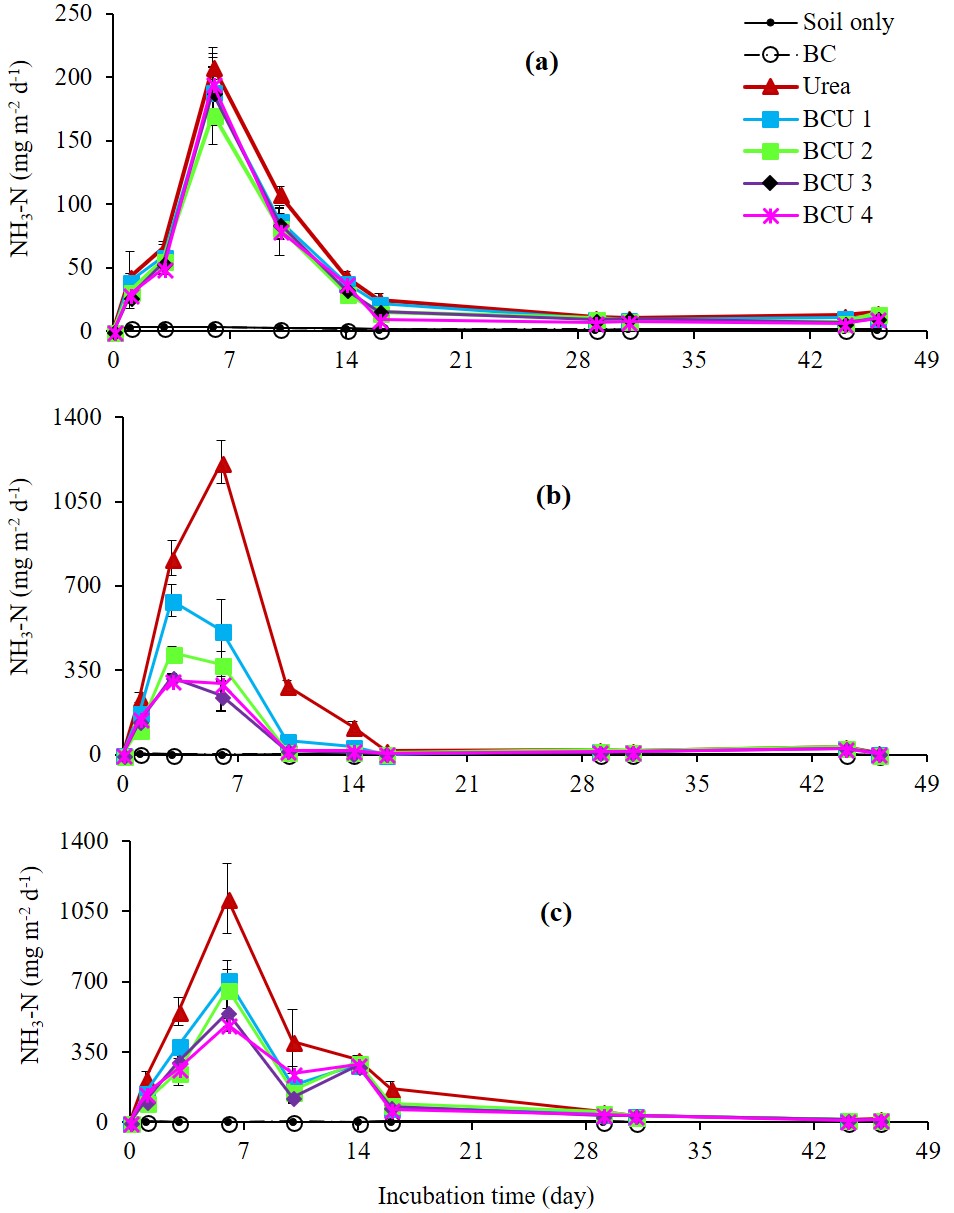


Figure S4. Daily NH_3_-N emissions from Ferrosol (a), Tenosol (b) and Vertosol (c) (values are mean ± standard error, N = 5).

# Soil analysis

Soil pH was determined at a soil-to-water ratio of 1:5 (WP 80 Reference pH Meter, Anpros Phy LTD., Victoria, Australia). Mineral N was extracted from soils with 2 M KCl using a 1:2.5 soil: extractant ratio. The soil extracts were filtered through Advantech filter paper 42 prior to analysis for mineral N species. The mineral N concentrations of soil were also measured following colorimetric method used for leachate analysis. Potentially mineralizable nitrogen (PMN) was assessed as described by Waring and Bremner ^5^, modified by Stenberg, Johansson ^6^. Total C and N in soil was measured using a high-frequency induction furnace CHN analyser (Vario Micro Cube). The soils were finely ground using a mortar and pestle prior to C and N analysis. Phosphorous, exchangeable Ca, Mg, K, extractable S, Al, and DTPA Fe were measured according to the standard methods described by Rayment, Lyons ^7^. The total C, N, NO_3_^-^-N, extractable sulfur and aluminium, phosphorous and DTPA-iron content of Ferrosol were substantially higher in Ferrosol compared to other soils. In contrast, the exchangeable calcium content was considerably higher in Vertosol than other soil types. No remarkable differences were found in exchangeable magnesium and potassium content among the three soils used in this study.


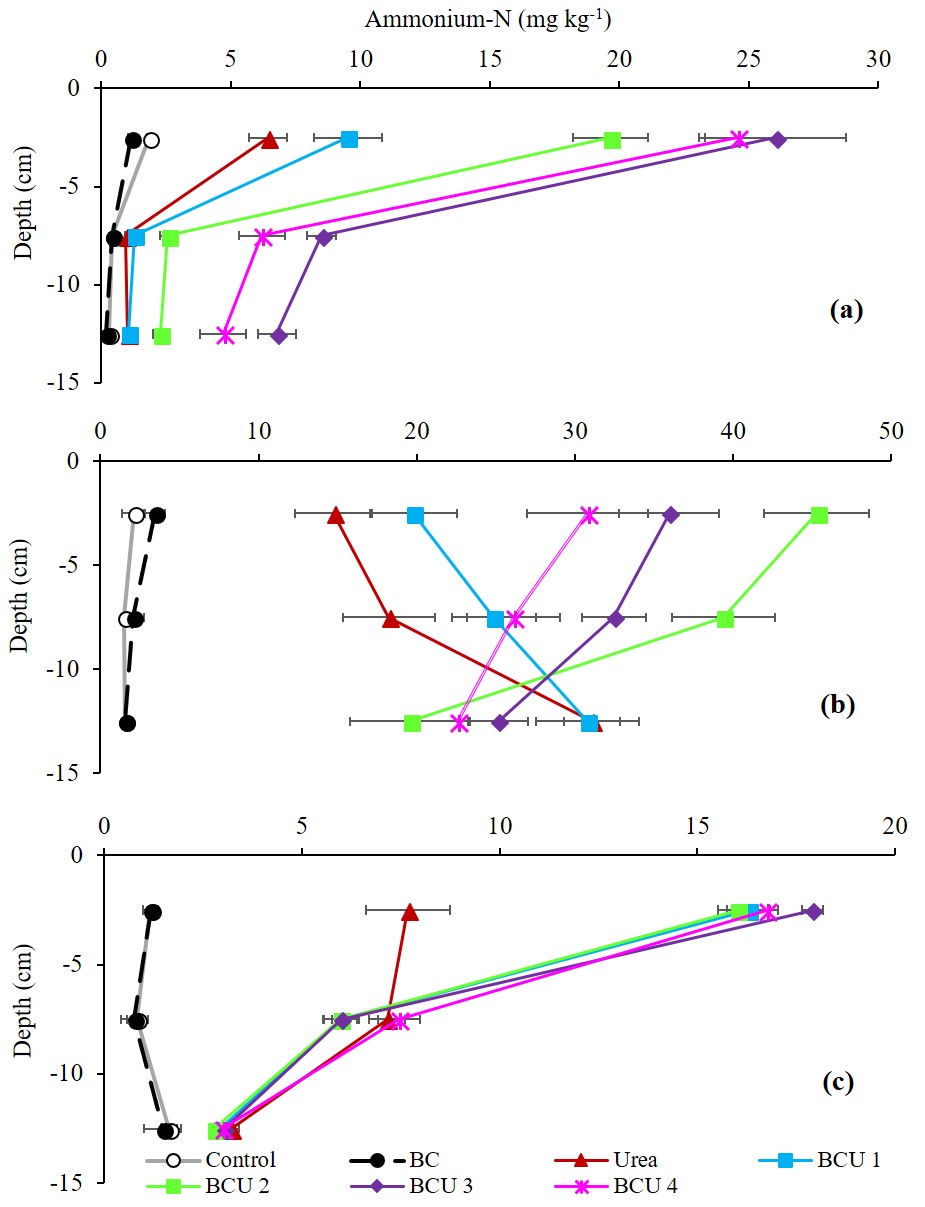


Figure S5. Ammonium-N content of soil profile at the end of incubation in a Ferrosol (a), Tenosol (b) and Vertosol (c) (values are mean ± standard error, N = 5).


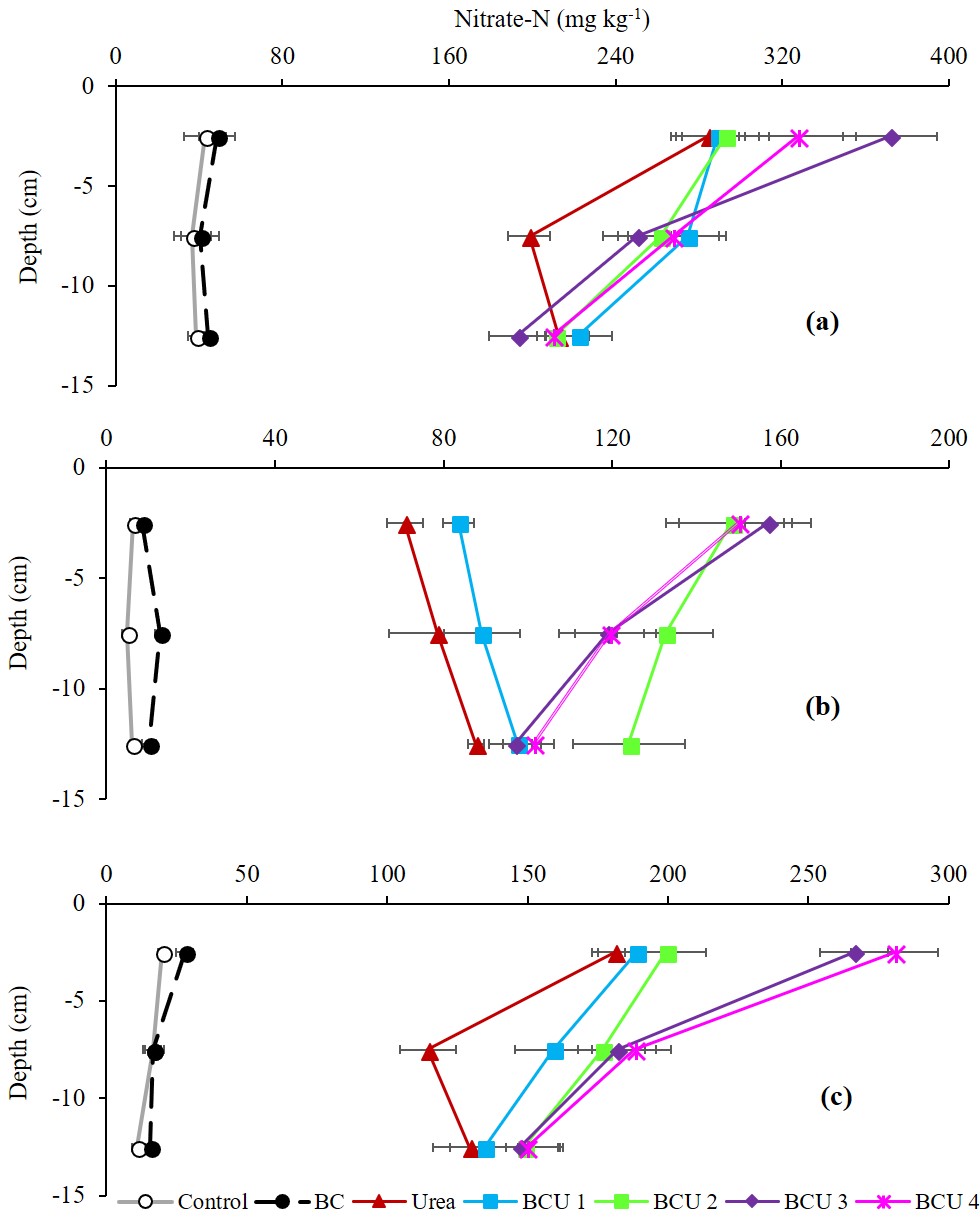


Figure S6. Nitrate-N content of soil profile at the end of incubation in a Ferrosol (a), Tenosol (b) and Vertosol (c) (values are mean ± standard error, N = 5).


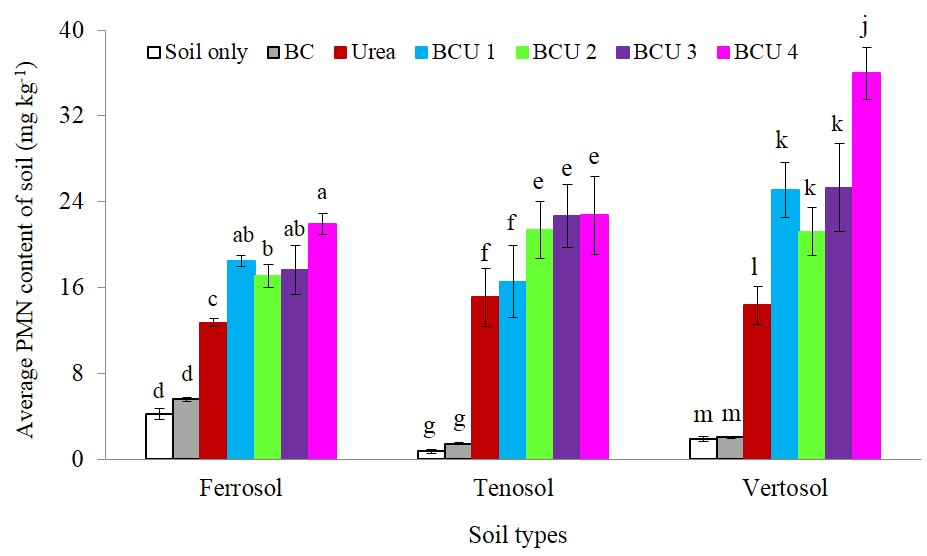
Figure S7. Average potentially mineralizable nitrogen (PMN) content of soil profile at the end of incubation in different soils (values are mean ± standard error, N = 5). Letters above columns are different if the values are significantly different (*P*<0.05). Valid statistical comparisons cannot be made between soil types.


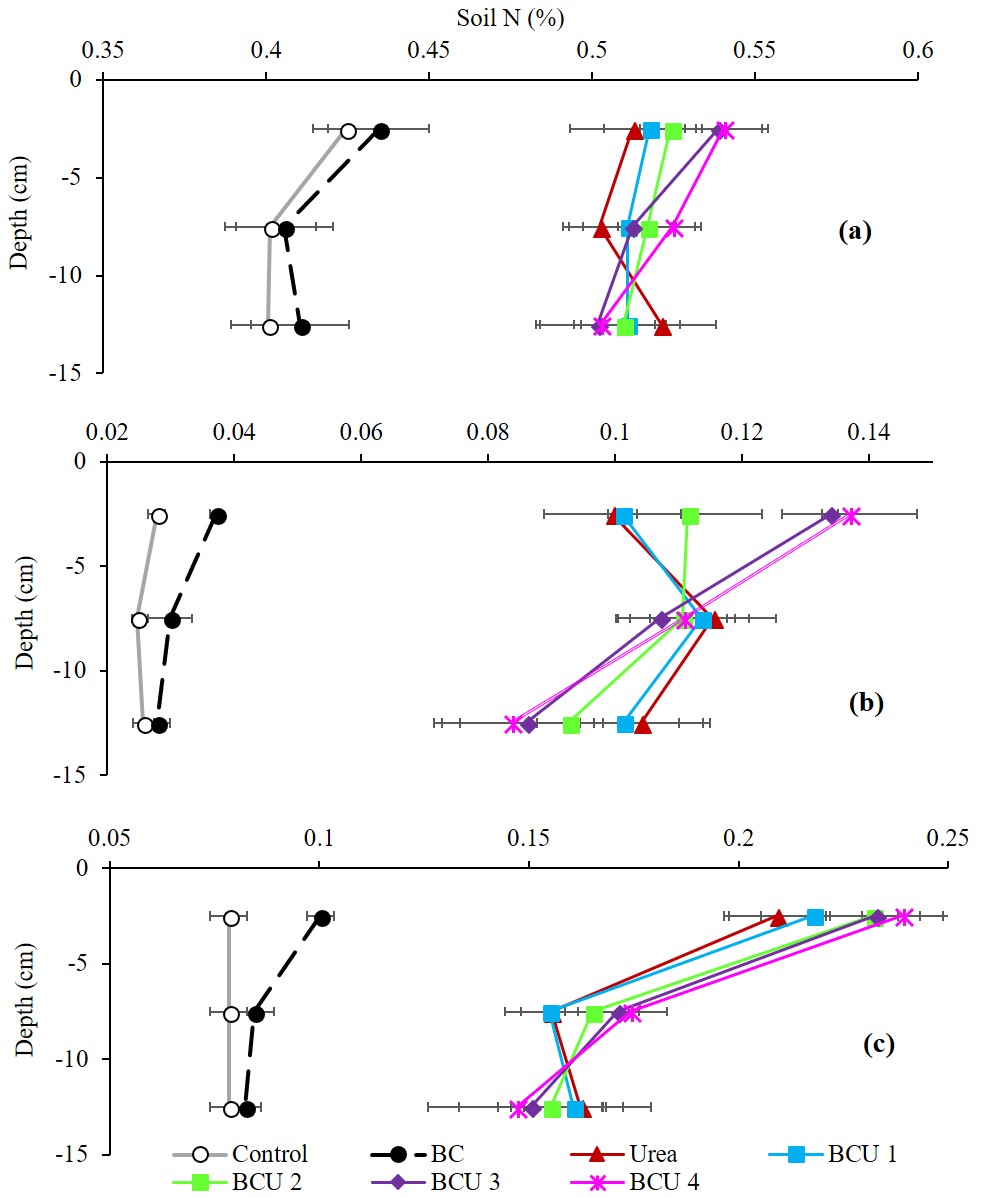


Figure S8. Total N concentration of soil profile at the end of incubation in Ferrosol (a), Tenosol (b) and Vertosol (c) (values are mean ± standard error, N = 5).

Table S2. Total C concentration of Ferrosol at various depth (values are mean ± standard error, N = 5).

| Treatments | Total C concentration of Ferrosol (%) | | |
| --- | --- | --- | --- |
|  | Top (0-5 cm) | Middle (6-10 cm) | Bottom (11-15 cm) |
| Control | 4.69±0.16c | 4.71±0.18a | 4.71±0.05a |
| BC | 6.01±0.14a | 4.97±0.07a | 4.73±0.08a |
| Urea | 4.79±0.05c | 4.79±0.06a | 4.72±0.04a |
| BCU 1 | 4.88±0.11bc | 4.73±0.18a | 4.73±0.08a |
| BCU 2 | 5.28±0.08bc | 4.96±0.06a | 4.78±0.04a |
| BCU 3 | 5.36±0.12b | 4.87±0.07a | 4.83±0.05a |
| BCU 4 | 5.99±0.16a | 4.93±0.08a | 4.83±0.06a |

Letters adjacent to values are different if the values within a column are significantly different (*P*<0.05).

Table S3. Total C concentration of Tenosol at various depth (values are mean ± standard error, N = 5).

| Treatments | Total C concentration of Tenosol (%) | | |
| --- | --- | --- | --- |
|  | Top (0-5 cm) | Middle (6-10 cm) | Bottom (11-15 cm) |
| Control | 0.79±0.03d | 0.67±0.03b | 0.74±0.04b |
| BC | 1.55±0.10a | 1.27±0.13a | 0.76±0.03b |
| Urea | 0.81±0.02d | 0.75±0.04b | 0.78±0.04b |
| BCU 1 | 1.06±0.07c | 0.93±0.13ab | 1.06±0.08a |
| BCU 2 | 1.18±0.03bc | 1.20±0.02a | 1.06±0.03a |
| BCU 3 | 1.17±0.03bc | 1.28±0.08a | 1.13±0.03a |
| BCU 4 | 1.42±0.15ab | 1.29±0.07a | 1.17±0.09a |

Letters adjacent to values are different if the values within a column are significantly different (*P*<0.05).

Table S4. Total C concentration of Vertosol at various depth (values are mean ± standard error, N = 5).

| Treatments | Total C concentration of Vertosol (%) | | |
| --- | --- | --- | --- |
|  | Top (0-5 cm) | Middle (6-10 cm) | Bottom (11-15 cm) |
| Control | 1.38±0.03e | 1.36±0.03c | 1.34±0.03b |
| BC | 2.07±0.06a | 1.48±0.02b | 1.45±0.04a |
| Urea | 1.45±0.02e | 1.39±0.03bc | 1.36±0.03b |
| BCU 1 | 1.58±0.05d | 1.43±0.03b | 1.49±0.03a |
| BCU 2 | 1.60±0.02cd | 1.46±0.02b | 1.46±0.03a |
| BCU 3 | 1.64±0.03c | 1.51±0.04a | 1.54±0.03a |
| BCU 4 | 1.88±0.04b | 1.47±0.02b | 1.53±0.02a |

Letters adjacent to values are different if the values within a column are significantly different (*P*<0.05).

# Post-harvest soil pH

Addition of BCU blends significantly affected the top soil pH at the end of incubation in all three soils (Table S5). Incorporation of BCU blends significantly lowered the post-harvest top soil pH compared to urea and control treatments in all the soil types. Overall, the higher the amount of BC in the blends, the lower the soil pH irrespective of soil types.

Table S5. Effect of BCU blends addition on the top (0-5 cm) soil pH at the end of incubation (values are mean ± standard error, N = 5).

| Treatments | Ferrosol | | Tenosol | | Vertosol | |
| --- | --- | --- | --- | --- | --- | --- |
|  | Initial | Post-harvest | Initial | Post-harvest | Initial | Post-harvest |
| Control | 4.69 | 4.71±0.01a | 7.24 | 7.19±0.02a | 8.32 | 8.30±0.01a |
| BC | 4.69 | 4.41±0.01b | 7.24 | 6.12±0.01d | 8.32 | 7.65±0.02f |
| Urea | 4.69 | 4.39±0.01b | 7.24 | 6.65±0.02b | 8.32 | 8.04±0.02b |
| BCU 1 | 4.69 | 4.32±0.02c | 7.24 | 6.46±0.02c | 8.32 | 7.98±0.01c |
| BCU 2 | 4.69 | 4.33±0.01c | 7.24 | 6.42±0.02c | 8.32 | 7.93±0.02d |
| BCU 3 | 4.69 | 4.35±0.02c | 7.24 | 6.41±0.03c | 8.32 | 7.75±0.02e |
| BCU 4 | 4.69 | 4.31±0.03c | 7.24 | 6.38±0.03c | 8.32 | 7.71±0.02e |

Letters adjacent to values are different if the values within a column are significantly different (*P*<0.05).

Table S6. *P*-value results from one-way ANOVAs for response variables

| Studied parameters | Ferrosol | Tenosol | Vertosol |
| --- | --- | --- | --- |
| Soil NH_4_^+^-N (0-5 cm) | 0.000 | 0.000 | 0.000 |
| Soil NH_4_^+^-N (6-10 cm) | 0.000 | 0.000 | 0.000 |
| Soil NH_4_^+^-N (11-15 cm) | 0.000 | 0.000 | 0.000 |
| Soil NO_3_^-^-N (0-5 cm) | 0.000 | 0.000 | 0.000 |
| Soil NO_3_^-^-N (6-10 cm) | 0.000 | 0.000 | 0.000 |
| Soil NO_3_^-^-N (11-15 cm) | 0.000 | 0.000 | 0.000 |
| Soil PMN (0-5 cm | 0.000 | 0.000 | 0.000 |
| Soil PMN (6-10 cm) | 0.000 | 0.003 | 0.000 |
| Soil PMN (11-15 cm) | 0.000 | 0.220 | 0.000 |
| Soil total N (0-5 cm) | 0.000 | 0.000 | 0.000 |
| Soil total N (6-10 cm) | 0.000 | 0.000 | 0.000 |
| Soil total N (11-15 cm) | 0.000 | 0.000 | 0.000 |


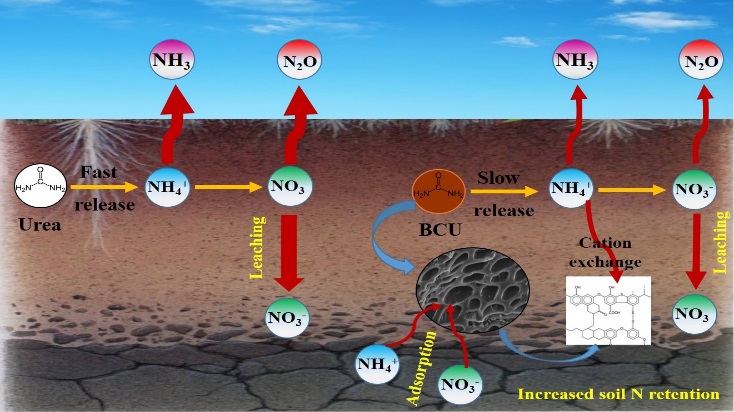


Figure S9. Mechanistic understanding of the effect of BCU and urea on the N dynamics

**REFERENCES**

1. Forster JC. Chapter 3 - Soil sampling, handling, storage and analysis. In: *Methods in Applied Soil Microbiology and Biochemistry* (ed^(eds Nannipieri KA). Academic Press (1995).

2. Miranda KM, Espey MG, Wink DA. A tapid, simple spectrophotometric method for simultaneous detection of nitrate and nitrite. *Nitric Oxide* **5**, 62-71 (2001).

3. Van Zwieten L*, et al.* Influence of biochars on flux of N_2_O and CO_2_ from Ferrosol. *Aust J Soil Res* **48**, 555-568 (2010).

4. Singh J, Saggar S, Bolan NS. Influence of dicyandiamide on nitrogen transformation and losses in cow-urine-amended soil cores from grazed pasture. *Anim Prod Sci* **49**, 253-261 (2009).

5. Waring SA, Bremner JM. Ammonium production in soil under waterlogged conditions as an index of nitrogen availability *Nature* **201**, 951-952 (1964).

6. Stenberg B, Johansson M, Pell M, Sjödahl-Svensson K, Stenström J, Torstensson L. Microbial biomass and activities in soil as affected by frozen and cold storage. *Soil Biol Biochem* **30**, 393-402 (1998).

7. Rayment GE, Lyons DJ, Shelley B. Soil Chemical Methods : Australasia. CSIRO publishing, Victoria, Australia.(2010).
